# On-Demand Design of Tunable Complete Photonic Band Gaps based on Bloch Mode Analysis

**DOI:** 10.1038/s41598-018-32422-1

**Published:** 2018-09-24

**Authors:** Shuo Li, Han Lin, Fei Meng, David Moss, Xiaodong Huang, Baohua Jia

**Affiliations:** 1 Centre for Micro-Photonics, Faculty of Science, Engineering and Technology, Swinburne University of Technology, Melbourne, VIC 3122 Australia; 2 Faculty of Science, Engineering and Technology, Swinburne University of Technology, Melbourne, VIC 3122 Australia; 3 State Key Laboratory of Advanced Design and Manufacturing for Vehicle Body, Hunan University, Changsha, 410082 China; 4 School of Civil Engineering, Central South University, Changsha, 410075 China

## Abstract

The fundamental property of photonic crystals is the band gap effect, which arises from the periodic dielectric modulation of electromagnetic waves and plays an indispensable role in manipulating light. Ever since the first photonic-bandgap structure was discovered, the ability to tune its bandgap across a wide wavelength range has been highly desirable. Therefore, obtaining photonic crystals possessing large on-demand bandgaps has been an ever-attractive study but has remained a challenge. Here we present an analytical design method for achieving high-order two-dimensional photonic crystals with tunable photonic band gaps on-demand. Based on the Bloch mode analysis for periodic structures, we are able to determine the geometric structure of the unit cell that will realize a nearly optimal photonic band gap for one polarization between the appointed adjacent bands. More importantly, this method generates a complete bandgap for all polarizations, with frequencies tuned by the number of photonic bands below the gap. The lowest dielectric contrast needed to generate a photonic band gap, as well as conditions for generating complete bandgaps, are investigated. Our work first highlights the systematic approach to complete photonic band gaps design based on Bloch mode analysis. The physical principles behind our work are then generalized to other photonic lattices.

## Introduction

Structures with periodic dielectric distributions, such as photonic crystals (PhCs), can achieve unique dispersion properties for controlling electromagnetic waves. Photonic band gaps (PBG), being used for wave confinement, are recognized as the most important feature of periodic structures. Their technological potential has enabled a wide scope of optical components such as waveguides and high-quality-factor resonators, where sizeable PBGs are highly demanded^1–3^. Therefore, it is of great importance to design PhCs with maximum PBG sizes to implement functional photonic devices with a desired performance. In addition, in most applications a complete photonic band gap (CPBG) at a targeted frequency range (e.g., the telecommunication window) is preferred, owing to the resulting capability of controlling all polarizations, e.g., the transverse electric (TE) and transverse magnetic (TM) polarizations in 2D cases.

Conventional design methods for PhCs directly employ regular lattices taken from nature (e.g., square, triangular or honeycomb etc.) that allow only one resonator in each unit cell^4–6^. As a result, the position and size of the resulting PBGs are not fully controllable. Meanwhile, numerical optimization methods that iteratively optimize the geometry with feedback from the calculated band diagram in each loop have been developed to obtain structures with PBG/CPBGs^7–9^. However, no systematic approach to CPBG design has been reported yet. Without any physical constrains, the resulting structures strongly depend on the randomly selected initial structures^8,10^. Therefore, a particularly desired PBG/CPBG can only be obtained through a large number of trials. Moreover, the control of CPBG still remains challenging due to the inevitable crosstalk between TE and TM polarization states.

It is of great interest to develop an analytical method to design PhC structures that can generate “on-demand” PBGs/CPBGs. Here we develop simple physical rules to design PBGs based on the Bloch mode analysis for periodic structures, suggesting an approach that harnesses complete band gap for all polarizations, with positions tuned by the number of photonic bands below the gap. Our approach directly links three key geometric parameters, namely the number, position, and geometric shape of the resonators, to the PBG properties of PhCs. In this way, the method is able to design structures with nearly optimal PBG sizes between arbitrarily appointed adjacent photonic bands for both TM and TE polarizations without any iterative calculations. More importantly, this method allows one to analytically design PhCs with tunable CPBGs, the position of which is controlled by the number of photonic bands below the gap. With this method, we are able to realize nearly optimal PhC structures composed of two arbitrary materials with dielectric constants *ε*_1_ and *ε*_2_. The required lowest dielectric contrast *ε*_2_/*ε*_1_ to generate the PBGs/CPBGs is also investigated.

**Figure 1:**
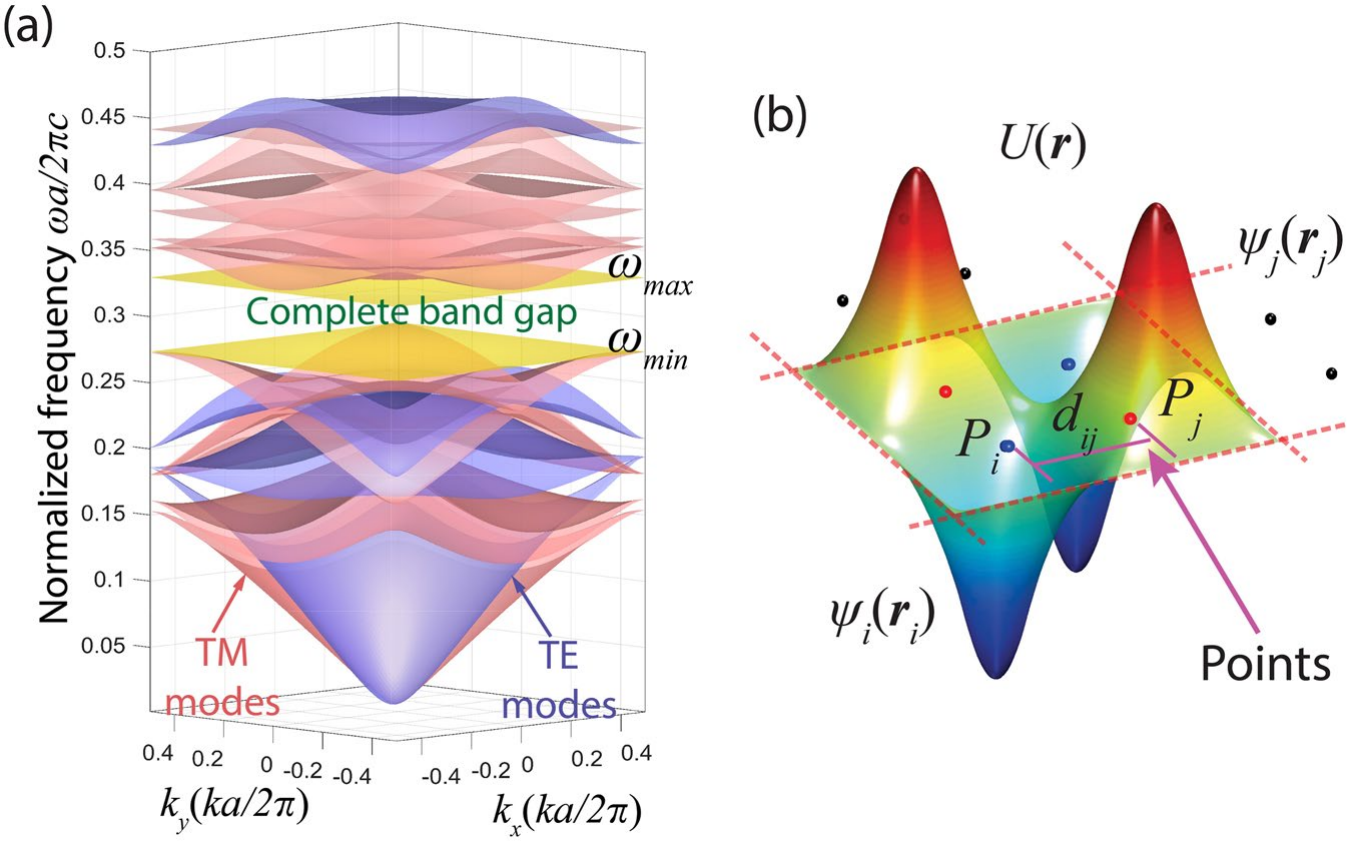
(**a**) A band diagram showing a CPBG in a square-latticed PhC. The CPBG is between the 3^rd^ and 4^th^ TE bands and the 5^th^ and 6^th^ TM bands. (**b**) A typical field distribution example of the Bloch mode in the unit cell of a high-order TM-PBG PhC with 4 rods positioned at the points.

Although the proposed method is applicable to any 2D lattice, here we consider the example of a 2D square lattice PhC in order to illustrate the process of our design method. The band diagram of a 2D PhC is depicted in Fig. 1(a). There is a TE-PBG between TE bands *N*_1_ and *N*_1_ + 1 and a TM-PBG between TM bands *N*_2_ and *N*_2_ + 1, respectively, where *N*_1_ and *N*_2_ denote the number of the photonic bands below the TE-PBG and TM-PBG separately. The CPBG exists due to the overlap of the TE-PBG and TM-PBG. Accordingly, two conditions are necessarily satisfied to obtain the structure that supports a large CPBG: 1) the ability to support both TE and TM PBGs and 2) achieving maximal overlap of them. With gap positions controlled by *N*_1_ and *N*_2_, our work in this paper provides an analytical method for the design of PhC structures with the maximum TE-PBG, TM-PBG and CPBG.

The size of CPBG can be expressed as follows:

1 $$\frac{{\rm{\Delta }}\omega }{{\omega }_{0}}=2\frac{\min ({\omega }_{{N}_{1}+1}^{TE},{\omega }_{{N}_{2}+1}^{TM})-\,\max ({\omega }_{{N}_{1}}^{TE},{\omega }_{{N}_{2}}^{TM})}{\min ({\omega }_{{N}_{1}+1}^{TE},{\omega }_{{N}_{2}+1}^{TM})+\,\max ({\omega }_{{N}_{1}}^{TE},{\omega }_{{N}_{2}}^{TM})}$$

where Δ*ω* and *ω*_0_ are the frequency range and the central frequency of the CPBG, respectively and *ω* is the eigenfrequency solved with the Maxwell equations^11^. In this work, *ω*_0_ represents the position of the CPBG that is controlled by the band order *N*_1_ and *N*_2_. For PBGs of only one polarization (TE/TM), Equation (1) can be simplified to $\frac{{\rm{\Delta }}\omega }{{\omega }_{0}}=2\frac{\min ({\omega }_{N+1})-\,\max ({\omega }_{N})}{\min ({\omega }_{N+1})+\,\max ({\omega }_{N})}$. Equation (1) provides the general characterization of the PBG size of PhC versus the wave vector ***k***, for both direct and indirect PBGs. Compared with the conventional square-latticed structures with a PBG above band 1, our designed PhCs with multiple bands below the first gap can be regarded as high-order PhCs.

Since Bloch’s theorem describes the field distributions of waves in periodic structures, the Bloch mode analysis has been widely applied in studying the performance of the existing periodic structures^12–14^. However, it has not been directly used in the design of geometric structures to achieve on-demand optical properties. The Maxwell equations in photonic crystals can be developed into the format of the standard wave equation ${\boldsymbol{\psi }}\text{'}\text{'}+A\cdot {\boldsymbol{\psi }}=0$, where **ψ** can be the electric field **E** or magnetic field **H**. In a 2D case, the nodes in the wave function are presented as nodal lines, and the antinodes can be defined as the points where the field amplitude achieves the maxima. So based on the criterion that the number of bands *N* equals to the number of antinodes of the Bloch mode with max (*ω*_*N*_) at M or Γ, we can design high-order photonic crystals by locating the resonators on the antinodes. Points *P* (e.g., *P*_*i*_ and *P*_*j*_ labeled in Fig. 1(b)) satisfy the following two conditions: 1) the points are the possible positions for a Bloch wave in order to generate antinodes for the electromagnetic field; 2) the points are the central positions of the resonators in the real space. The Bloch mode of a high-order PhC can be treated as the super-mode formed by the resonant states at each point. Figure 1(b) presents the schematic of the Bloch mode in a unit cell in a periodic structure, which is the eigenvector at a certain eigenfrequency *ω* (**E** field for the TM modes and **H** field for the TE modes). It can be expressed as a linear combination of Bloch waves *ψ*_*i*_ (***r***)^15^:

2 $$U({\boldsymbol{r}})=\sum _{i=1}^{N}{c}_{i}{\psi }_{i}({\boldsymbol{r}}){e}^{i{\phi }_{i}}$$

where *c*_*i*_ is the weighting coefficient for each Bloch wave, *φ*_*i*_ and ***r*** are the phase term and the position vector, respectively.

## Material and Methods

### Band Order

On the basis of previous theoretical studies^11,16^ resonators need to be high dielectric constant rods in order to yield TM-PBG structures performing as the Mie resonators, and low dielectric constant holes in order to yield TE-PBG structures to enable the Bragg-like multiple scattering. In the unit cell of high-order PhC, the Bloch modes with frequencies below the first gap are formed by the fundamental resonant state at each point. Assuming all the points *P* inside the unit cell are identical and the wave function *ψ*_*i*_ at each point is the same, the Bloch wave can be expressed as a linear combination of waves at each point, as shown in Equation (2). Each state comprises one Bloch state and behaves as one Bloch mode in the photonic band diagram, corresponding to a certain band^17^. Therefore the total number of Bloch modes is^18^:

3 $${{\rm{\Omega }}}_{Bloch}=\frac{[N+(2-1)]!}{N!(2-1)!}-1=N$$

The number of bands *N* equals the number of antinodes of the Bloch mode $\min ({\omega }_{{N}_{1}+1}^{TE},{\omega }_{{N}_{2}+1}^{TM})$ at M or Γ (Supplementary Appendix A). Therefore, we can draw the conclusion that in order to generate *N* bands below the gap, one requires *N* points in the unit cell.

**Figure 2:**
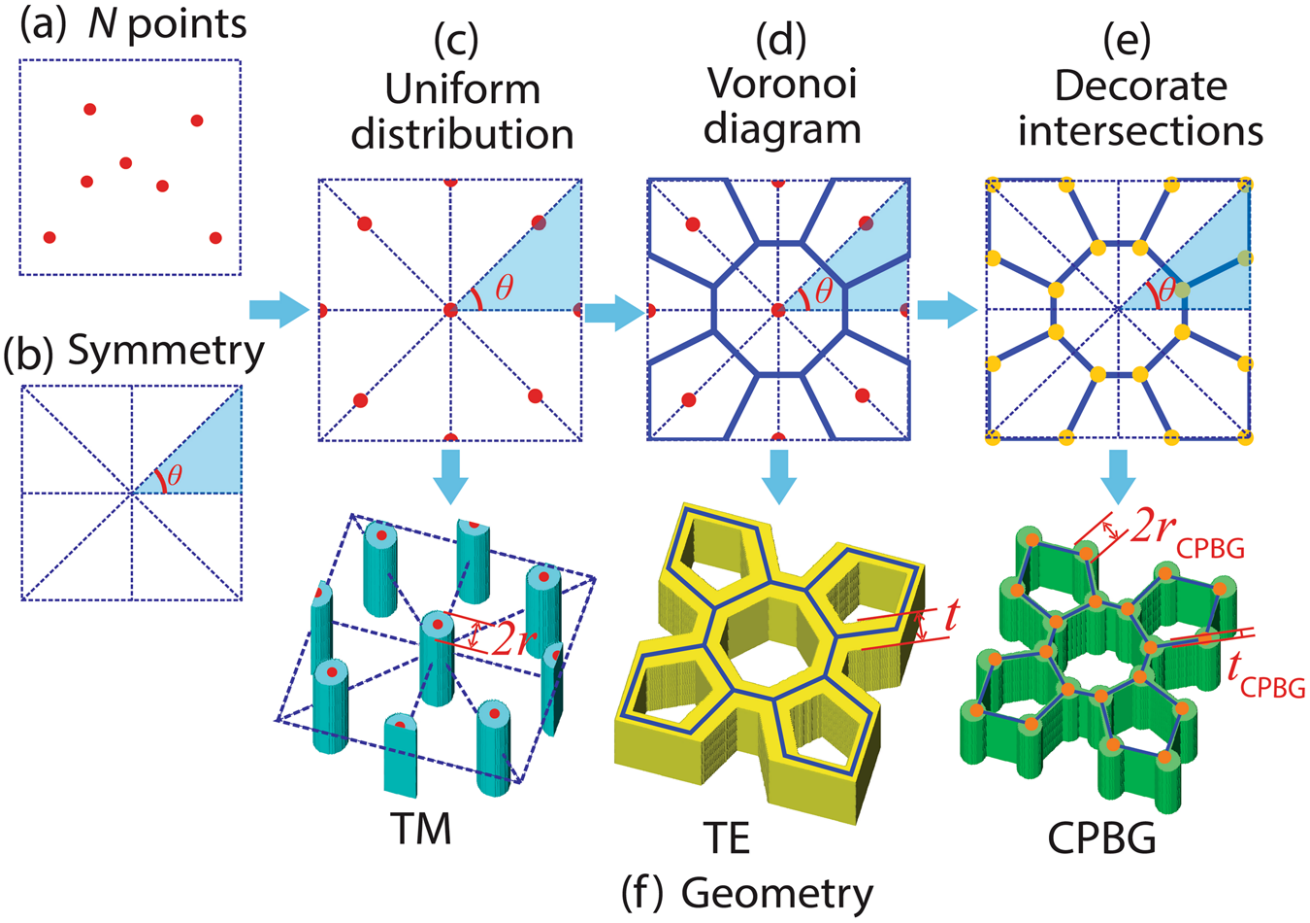
Design process to generate a band gap above band *N* (*N* = 7 for example). Number of points, symmetry and distribution are designed following (**a**–**e**). PhC structures that support TM-PBG, TE-PBG and CPBG are achieved showing in (**f**).

For a given number *N*, the control of the gap position and the design of the targeted structures with a PBG between band *N* and *N* + 1 can be realized by our proposed method. The count of the total number of points following: the points inside the Brillouin zone is counted as 1, on the edges of the Brillouin zone it is 0.5 and at the corners it is 0.25. In Fig. 2 there are 5 points inside the squared Brillouin zone and 4 on the edges, therefore the total number of points are 7. The process is illustrated in Fig. 2, where we analytically design step by step, the spatial distribution of points *P* and the shape of each resonator that determines the ultimate structures of the 2D PhCs.

### Spatial Distribution

The spatial distributions of points *P* in a certain unit cell are controlled by two factors: 1) the rotational symmetry constraint determined by the Brillouin zone; and 2) uniform distribution of these points. For a square-latticed PhC, *C*_4_ rotational symmetry is required (Fig. 2(b)). Meanwhile, a uniform spatial distribution of the points is also desired to attain a minimal phase mismatch of Bragg conditions in all directions and the strongest local confinement of resonances at each point size, thereby a large band gap can be achieved. The uniformity can be characterized by the distance between the neighbouring points. For a certain point *P*_*i*_ (*i = *1, 2,…, *N*) in Fig. 1(b), assuming that there are *x*_*i*_ neighbour points *P*_*j*_ (*j = *1, 2,…, *x*_*i*_), the deviation of the neighbour distances from the uniform distribution can be expressed as^19^

4 $$\delta =\sum _{i=1}^{N}\sqrt{\sum _{j=1}^{{x}_{i}}{({d}_{ij}-a)}^{2}/{x}_{i}}/Na$$

where *a* is the lattice constant and *d*_*ij*_ denotes the distance between points *P*_*i*_ and *P*_*j*_. To ensure uniform distribution (Fig. 2(c)), the minimal *δ* should be achieved. Equation (4) quantifies the deviation of the spatial distribution from the uniform distribution, and finally influences the size of PBG (Supplementary Appendix B). As a result, the positions of the resonators at these points can be defined by Equation (4).

### Geometry

Subsequently, we design the geometric shape of each resonator in order to optimize the resulting band structure. According to the electromagnetic variational theorem^11^, confining the electric field **E** within regions with a high dielectric constant minimizes the physical energy of the mode. Therefore, the geometric structures of high permittivity material are determined by the **E** field distribution of the Bloch mode. TM mode possesses an **E** field perpendicular to the periodic plane, thus the structures supporting the TM-PBG should be composed of isolated high permittivity rods locating on the points, performing as the Mie resonators. On the other hand, for TE polarization with the magnetic field **H** perpendicular to the periodic plane, the **E** field reaches a maximum on the zero-intensity region of **H** field according to ${\bf{E}}({\boldsymbol{r}})=\frac{i}{\omega {\varepsilon }_{0}\varepsilon ({\boldsymbol{r}})}\nabla \times {\bf{H}}({\boldsymbol{r}})$^11^. In this case, the high permittivity area should be in the shape of connected walls, which can be analytically calculated via the Voronoi diagram generated by the *N* points (Fig. 2 (d)). Each partition of the Voronoi diagram operates as a resonator to generate the local resonant states of **H** field. The actual geometry of both the rods and the Voronoi structures are determined by the filling ratio of the high permittivity material that influences the size of the PBG.

For simplicity we adopt circular rods that are of identical size and connected walls that are of identical thickness. In the 2D PhC the normal-incidence gap along one direction reaches a maximum when there are same optical path lengths in each material^11^. Therefore, the optimal radii of the rods *r* and the thickness of the walls *t* satisfy:

5 $$r,t=\frac{\sqrt{{\varepsilon }_{1}}}{\sqrt{{\varepsilon }_{2}}+\sqrt{{\varepsilon }_{1}}}\cdot \frac{\bar{d}}{2}=\frac{\sqrt{{\varepsilon }_{1}}}{\sqrt{{\varepsilon }_{2}}+\sqrt{{\varepsilon }_{1}}}\cdot \frac{{\sum }_{i=1}^{N}{\sum }_{j=1}^{N}{d}_{ij}}{2N}$$

where $\bar{d}$ is the average neighbour distance of the points *P*. With the optimal *r*, *t* in Equation (5) and the minimal *δ* in Equation (4), the actual geometry of the 2D PhCs for a nearly optimal PBG can be designed analytically.

## Results and Discussion

### Band gap results

**Figure 3:**
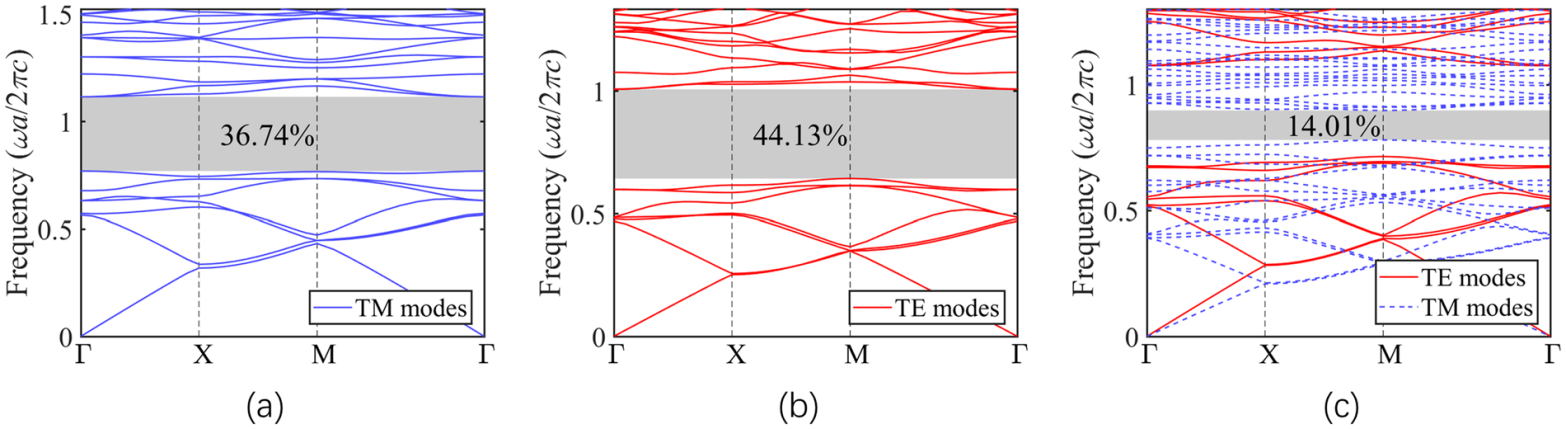
Band diagrams corresponding to the designed PhC structures in Fig. 2. (**a**) Band diagram of *N* = 7 TM structure (*ε*_2_ = 12, *ε*_1_ = 1). (**b**) Band diagram of *N* = 7 TE structure (*ε*_2_ = 12, *ε*_1_ = 1). (**c**) Band diagram of *N*_*1*_ = 7, *N*_2_ = 13 complete band gap structure (*ε*_2_ = 20, *ε*_1_ = 1).

**Figure 4:**
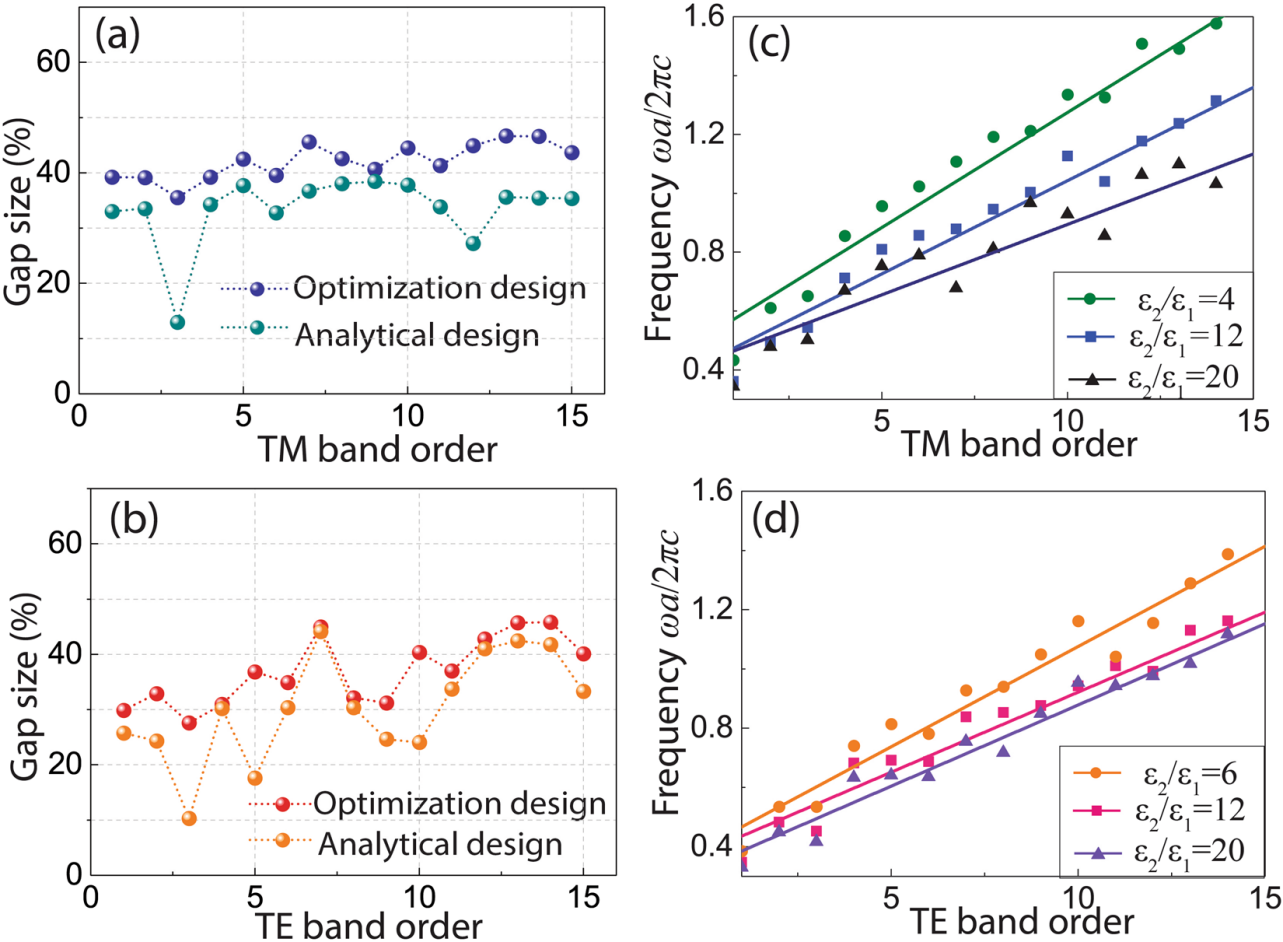
(**a**,**b**) Gap sizes of the analytical designs and optimization designs for (**a**) TM and (**b**) TE polarizations, respectively (*ε*_2_ = 12, *ε*_1_ = 1). (**c**,**d**) Relation of the central frequencies *ω*_0_ and TM/TE PBG orders. The shaded areas in (**c**,**d**) represent the results for different dielectric contrasts.

The designed structures for TM-PBG and TE-PBG when *N* = 7 are presented in Fig. 2(f) as an example. Finite element method (FEM) is employed to calculate the PBGs. The band diagrams corresponding to the designed PhCs in Fig. 2 (f) are shown in Fig. 3. The resulting PBGs of the analytically designed TM and TE structures with *N* from 1 to 15 (*ε*_2_ = 12, *ε*_1_ = 1) are shown in Fig. 4(a,b). The results indicate that the analytical design method is capable to achieve large PBGs for any number of *N*, thus validating the generality of the proposed method.

To verify the designed PBGs achieve a nearly optimum band gap size, the designed structures is further optimized by using the bi-directional evolutionary structural optimization (BESO) method^20,21^ to refine the geometric shapes (Supplementary, Appendices C & D). It is interesting to note that by using our simplified design method, the iterations required for the BESO program to obtain the optimized results is only half of the calculation loops for the BESO with random initial structures. The final designs of the optimized structures have the same topology as our design since we set our design as the initial structures of the optimization function. More importantly, better PBG sizes can be achieved due to the fine tuning of the shape of each resonator. By combining our analytical analysis with the BESO method, not only is the direct physical connection between structure topology and photonic band gaps revealed but also are the near optimal structures achieved, making the optimized structures physically meaningful for the design and implementation of PhC devices. The optimized PBG sizes are also shown in Fig. 4(a,b), which are slightly larger than the analytical designs due to the fine tuning of the shape of each resonator after the optimization iterations. The average discrepancy between the designed and the optimized gap sizes is calculated by ${\sum }_{n=1}^{N}|Ga{p}_{{\rm{design}}}-Ga{p}_{{\rm{optimization}}}|/N$, which is 8.59% for TM polarization and 6.26% for TE polarization, respectively. Such small discrepancies indicate that our analytical designs have achieved nearly optimal results. In our design method the symmetry and the distribution uniformity can be predicted, but the final geometry can be only estimated simply by the rod radius *r* or the Voronoi wall thickness *t*, as shown in Equations (5) and (6). However, the geometry of the optimized structure can be much more complex with different *r or t* in different directions. Therefore, the discrepancy comes from the geometry difference between the analytical design and the optimized one.

Moreover, it is also clearly seen that optimized high-order designs show larger PBG sizes comparing with the low-order ones because of the nearly uniform distribution that high-order ones can achieve. It was suggested that lattices with a higher rotational symmetry can achieve larger gap sizes due to increased symmetry^22^. In our design process, square lattice was demonstrated for simplicity. But the principle is expected to work the same for other lattices, since the design theory is not dependent on the lattice type. In addition, this physical principle can also well explain the larger bandgaps hosted in hexagonal lattices, which is attributed to the higher rotational symmetry, further confirming our symmetry criterion.

Increasing the number of bands below the gap results in a blue shift of PBG position and thus the high-order designs make tuning of the gap position possible. Since the frequencies in the simulation is normalized and using the lattice constant *a* as a measure of scale, the band diagram is readily scalable for any wavelength. Based on the designed structures, Fig. 4(c,d) show the dependence of the normalized central frequencies *ω*_0_ of the gaps on the TM/TE band order, *N* (optimized structures). The linear dependence indicates that the position of the gap can be tuned by changing *N*. Figure 4 (c,d) also provide the *ω*_0_ − *N* results under different dielectric contrast *ε*_2_/*ε*_1_. It shows the feasibility to tune the position of the PBG under any dielectric contrast, which can perform as an atlas to design PBG for a specific dielectric contrast in a desired frequency range. For example, if a TM band gap with central frequency at communication wavelength 1.55 μm is targeted, for a lattice constant of 1.5 μm. In Fig. 4 (c) there are several structures around *ω*_0_ = 1 with different material indices and band orders to harness bandgaps, e.g. band order 6 with *ε*_2_/*ε*_1_ = 4, band order 9 with *ε*_2_/*ε*_1_ = 12, band order 9 & 14 with *ε*_2_/*ε*_1_ = 20. Other targeted wavelengths and material indices can be predicted in the same way with the maps in Fig. 4 (c,d).

To design structures with CPBG, usually the parametric studies with physical reasoning face the difficulty to meet the different structure requirements of TE and TM polarizations, resulting in uncontrollable band gap sizes. Meanwhile, topology optimization approaches in general suffer from low regularity due to the presence of multiply eigenvalues. Based on our achieved nearly optimal TE/TM-PBG structures, further design of the structures with large CPBGs can be conducted. Direct combination of the TM-PBG and TE-PBG structures at the same order cannot lead to a CPBG due to the crosstalk of TE and TM modes induced by the mutual geometrical perturbations. Given the former designs, the TE-PBG structure is possible to support TM modes because the intersections of the walls can be regarded as rods (shown as yellow dots in Fig. 2(e)). Therefore, it is possible to control the geometric shape of the intersections in a TE-PBG structure in order to achieve a TM-PBG at the same time, and thus achieving a CBPG. We choose the radius *r*_CPBG_ to design the rods and the thickness of walls *t*_CPBG_ to tune the size and position of the gaps for both polarizations, and then make them overlap to generate the CPBG. Take the example of *N*_1_ = 7 in Fig. 2(e), there are 13 interconnections (*N*_2_ = 13) in the unit cell. Based on the former criteria, it is easy to predict that the TE-PBG is between band 7 and 8, and that the TM-PBG is between band 13 and 14.

Since the rods located at the intersections of the TE walls usually cannot achieve a uniform distribution, the design based on Equation (5) is no longer directly applicable. To solve this problem, we define the intersections of the TE walls, and then apply the minimal neighbour distance of these intersections, min(*d*_*ij*_) in the CPBG design. The radius of the rods in the CPBG design is

6 $${r}_{{\rm{CPBG}}}=\frac{\sqrt{{\varepsilon }_{1}}}{\sqrt{{\varepsilon }_{2}}+\sqrt{{\varepsilon }_{1}}}\cdot \frac{\min ({d}_{ij})}{2}$$

The value of the wall thickness, *t*_CPBG_, should be smaller than the rod diameter, 2*r*_CPBG_, aiming at minimizing the geometry perturbation to the TM PBG. In the design process, we find that CPBG structures can be generally achieved for *t*_CPBG_ around 0.06*a*.

**Figure 5:**
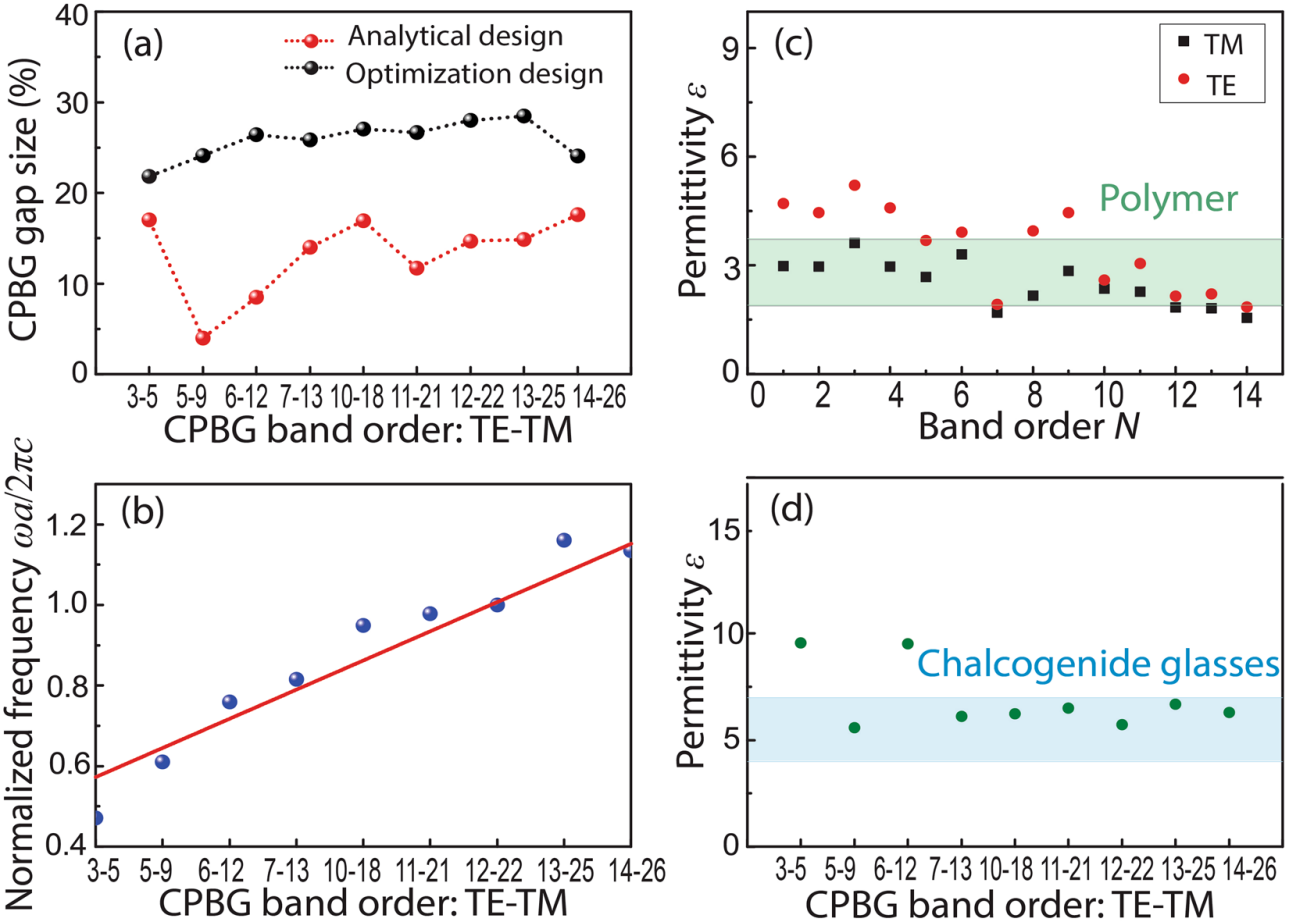
(**a**) CPBG sizes of analytical design (red) and topology optimization (black). (**b**) Relation of central frequency *ω*_0_ and CPBG order. The blue dots are the simulation results and the red line shows linear fitting. (*ε*_2_ = 20, *ε*_1_ = 1). (**c**,**d**) Minimal permittivity to generate the TM (black) and TE (red) PBGs (**c**) and a CPBG (**d**) above different band orders. Shaded area represents the permittivity range of polymers (green) chalcogenide glasses (blue).

Figure 5(a) presents the comparison between the analytical designs and the optimization results for the CPBG (*ε*_2_ = 20, *ε*_1_ = 1). A large permittivity was used to demonstrate under the same permittivity, large band gaps can be generated for all the orders *N*. The CPBG bands are arranged in an increasing order for the TE modes. It is noticeable that some orders are missing (TE-TM order: 1-1, 2-2, 4-4, 8–10 and 9–13) because there is no CPBG at these band order (Supplementary, Appendix E). The results show that the analytical designs can achieve large CPBGs without iterative calculations. The optimization process only helps to enlarge the CPBG sizes on the basis of the initial structures. Figure 5(b) depicts the relation between *ω*_0_ and the CPBG band order (*ε*_2_ = 20, *ε*_1_ = 1), indicating that the CPBG position can also be tuned by changing the number of bands below the gap. It is worth pointing out that the analytical designs approach presented are based on ideal 2D photonic crystals. For some practical applications with different boundary conditions—such as photonic crystal slabs, the results may not be straightforwardly applicable and additional topology optimization may be required.

### PBG/CPBG with Low Dielectric Contrast

With the optimal PhCs, we take a further step to explore the minimal dielectric contrast to generate a PBG/CPBG. Former studies have realized low-index band gap with quasi-structures^22^. Here we focus on periodical PhCs, which have low complexities in both calculation and fabrication. In contrast to a conventional PhC with only one resonator in a unit cell, high-order PhCs are able to generate large PBG at a low dielectric contrast. Owing to their higher uniform distribution, high-order PhCs can decrease the undulations of the frequency bands that suppress the gap size, leading to more isotropic gaps for light propagating in different directions. As a result, they are able to generate sizeable CPBGs for both TE and TM polarizations by using low-refractive index materials, which is hardly possible with conventional ones.

Taking the dielectric-air structure (*ε*_1_ = 1) as an example, we find that the minimal dielectric permittivity *ε*_min_ required to generate a PBG and a CPBG at different band orders, as shown in Fig. 5(c,d). In the high-order PhC designs, the minimal permittivity to generate a PBG can be as low as 1.55 for TM mode and 1.85 for TE mode (*N* = 14). Such values are much lower than the optimized structures with a fourfold rotational symmetry that generates PBGs with a dielectric contrast around 3^22^. It can be seen from Fig. 5(d) that the minimal permittivity to generate a CPBG can be significantly reduced to 5.59 (*N*_1_ = 5, *N*_2_ = 9), which, to the best of our knowledge, is the lowest dielectric contrast needed to generate a CPBG in the 2D case reported to date. This permittivity matches with the ranges of polymers and chalcogenide glasses (shaded areas in Fig. 5(c,d)), indicating that these high-order PhCs can be realized with these easy-fabricated materials^23,24^.

## Conclusions

In conclusion, we have developed an analytical design method for high-order PhC structures with nearly optimal CPBGs/PBGs free of iterative calculations. The design criteria are derived from the Bloch mode analysis and expressed by analytical mathematical equations. This study links the periodic structures to the physical properties of PBG analytically. Furthermore, the lowest dielectric contrasts needed in order to generate a PBG/CPBG have also been explored. The design principle can be applied to 2D PhCs with arbitrary lattices. The extension to 3D periodic structures could provide a better insight into the interaction between the geometry of structures and the electromagnetic waves. Regardless of the dimensionalities of photonic crystals, they are based on the Bloch theory. Therefore our method is expected to provide a potential solution for 3D photonic lattices, where conventional design methods based on the naturally available crystalline structures see a major challenge in achieving PBGs for all polarizations, in particular at low dielectric constants. Since a PBG is the most fundamental property of PhCs, our method is expected to form the basis of design and implementation of PhC devices, thereby opening up new ways to a number of PhC enabled applications.

## Electronic supplementary material

Supplementary Information

## Data Availability

All data generated or analysed during this study are included in this published article (and its Supplementary Information files).
