# Supplementary material for: On-Demand Design of Tunable Complete Photonic Band Gaps based on Bloch Mode Analysis
Source: Sci Rep. 2018 Sep 24;8:14283. doi: 10.1038/s41598-018-32422-1 (PMC6155245; doi:10.1038/s41598-018-32422-1)
Supplement: Supplementary file 1 — Supplementary Information [file 41598_2018_32422_MOESM1_ESM.pdf]

Supplementary Information for

On-Demand Design of Tunable Complete Photonic Band Gaps based on Bloch Mode Analysis

Shuo Li[1], Han Lin[1], Fei Meng[2,4], David Moss[1], Xiaodong Huang[2,3], and Baohua Jia[1]

[1]*Centre for Micro-Photonics, Faculty of Science, Engineering and Technology, Swinburne University of Technology, Hawthorn, VIC 3122, Australia*
[2]*Faculty of Science, Engineering and Technology, Swinburne University of Technology, Melbourne, VIC 3001, Australia*
[3]*State Key Laboratory of Advanced Design and Manufacturing for Vehicle Body, Hunan University, Changsha 410082, China*
[4]*School of Civil Engineering, Central South University, Changsha 410075, China*

Appendix A: Number of points and Bloch modes

It is well known that the Maxwell equations in the vacuum can be developed into the electromagnetic wave equation. The wave equation of a photonic crystal can be obtained in a similar way. In photonic crystal, the space is charge-free, and the materials are characterized with a scalar dielectric function $\varepsilon(\mathbf{r},\omega)$. So the Maxwell Equations are (1):

$$\nabla\cdot\varepsilon(\mathbf{r})\mathbf{E}=0 \tag{S1}$$

$$\nabla\times\mathbf{E}=-\mu_0\frac{\partial\mathbf{H}}{\partial t} \tag{S2}$$

$$\nabla\cdot\mathbf{H}=0 \tag{S3}$$

$$\nabla\times\mathbf{H}=\varepsilon_0\varepsilon(\mathbf{r})\frac{\partial\mathbf{E}}{\partial t} \tag{S4}$$

By taking the curl of Eqs. (S2) and (S4), we can obtain the Wave Equations:

$$\nabla\times\nabla\times\mathbf{E}-\frac{\varepsilon(\mathbf{r})}{c^2}\cdot\frac{\partial^2\mathbf{E}}{\partial t^2}=0 \tag{S5}$$

$$\nabla\times(\frac{1}{\varepsilon(\mathbf{r})}\nabla\times\mathbf{H})-\frac{1}{c^2}\cdot\frac{\partial^2\mathbf{H}}{\partial t^2}=0 \tag{S6}$$

where $c^2=\frac{1}{\varepsilon_0\mu_0}$. The plane waves that have the following format are one type of solutions of Eqs. (S5) and (S6):

$$\mathbf{E}(\mathbf{r},t)=\mathbf{E}(\mathbf{r})e^{-i\omega t} \tag{S7}$$

$$\mathbf{H}(\mathbf{r},t)=\mathbf{H}(\mathbf{r})e^{-i\omega t} \tag{S8}$$

To simplify the format of Eqs. (S5) and (S6), the differential operators $\widehat{\Theta}_1$ and $\widehat{\Theta}_2$ can be defined as:

$$\widehat{\Theta}_1\mathbf{E}(\mathbf{r})\triangleq\nabla\times\nabla\times\mathbf{E}(\mathbf{r}) \tag{S9}$$

$$\widehat{\Theta}_2\mathbf{H}(\mathbf{r})\triangleq\nabla\times(\frac{1}{\varepsilon(\mathbf{r})}\nabla\times\mathbf{E}(\mathbf{r})) \tag{S10}$$

$\widehat{\Theta}_1$ and $\widehat{\Theta}_2$ are the Hermitian operators and play an essential role to transform the Maxwell equations into an eigen problem. Then the wave equations can be expressed as the following format:

$$\widehat{\Theta}_1\,\mathbf{E}(\mathbf{r})-\left(\frac{\omega}{c}\right)^2\varepsilon(\mathbf{r})\mathbf{E}(\mathbf{r})=0 \tag{S11}$$

$$\widehat{\Theta}_2\,\mathbf{H}(\mathbf{r})-\left(\frac{\omega}{c}\right)^2\mathbf{H}(\mathbf{r})=0 \qquad \text{(S12)}$$

Eq. (S11) and Eq. (S12) follow the format of the standard wave equation:

$$\boldsymbol{\psi}''+A\cdot\boldsymbol{\psi}=0 \qquad \text{(S13)}$$

and $\boldsymbol{\psi}$ can be the electric field **E** or magnetic field **H**. Considering the complexity of the dielectric function $\varepsilon(\mathbf{r})$, it is impossible to provide the analytical expression of $\boldsymbol{\psi}$. And the solution of $k_x(\omega)$ and $k_y(\omega)$ are given by numerical calculations such as the plane wave expansion (PWE) method, finite-difference time-domain (FDTD) method and finite element method (FEM).

Based on the wave equations Eq. (S11) and Eq. (S12), we are able to explore the electromagnetic field based on the general case of how the wave behaves inside a periodic dielectric environment, instead of discussing **E** or **H** separately.

Based on the periodic boundary condition, the two components, k_x, k_y of the wave vector ***k*,** need to be quantized (2). For a square-latticed photonic crystal, the Brillouin zone in ***k***-spaces need to follow C_4 rotational symmetry, so k_x and k_y are reversible. Therefore the antinodes can be clearly observed at M or Γ with $k_x = k_y$. For ψ_f at each point P_i defined in the manuscript, the phase φ_i can be 0 or π. At the bottom frequency of the photonic band gap $\omega_{\min}$, the Bloch mode is the state that all the neighbor points are out of phase. Therefore the number of bands *N* equals to the number of antinodes of the Bloch mode of $\omega_{\min}$ at M or Γ.

In a 2D case, the nodes in the wave function $\psi = 0$ can be presented as the nodal lines instead of points in the 1D case. But the antinodes can still be defined as the points where the field amplitude achieves the maxima. So based on the criterion that the number of bands *N* equals to the number of antinodes of the Bloch mode with $\omega_{\min}$ at M or Γ, we can design high-order photonic crystals by locating the resonators on the antinodes.

The discussion above is based on the properties of waves in a periodic structure. We are able to observe this phenomenon in photonic crystals. Fig.S1 presents the Bloch modes with $\omega_{\min}$ at M or Γ for different high order photonic crystals. The antinodes can be clearly observed (E field for TM-PBG and H field for TE-PBG at *z* direction).

	Bloch Mode	Photonic crystal structure	Band diagram
(a)<br>TM-PBG,<br>Band 4 at<br>Γ,<br>Ez field	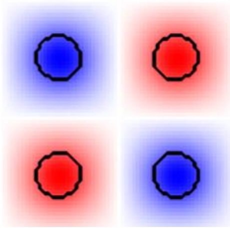	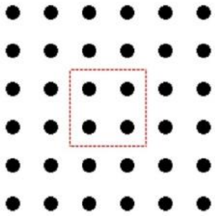	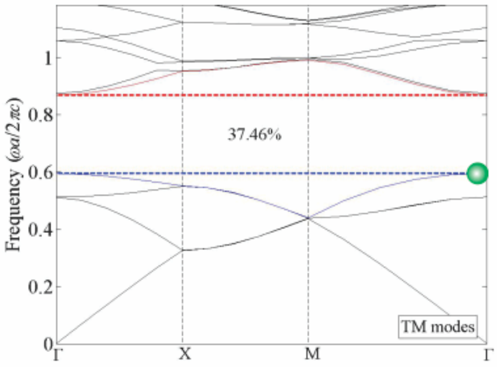
(b)<br>TM-PBG,<br>Band 5 at<br>M,<br>Ez field	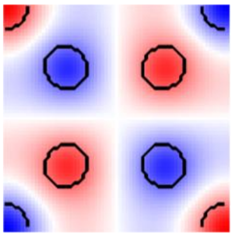	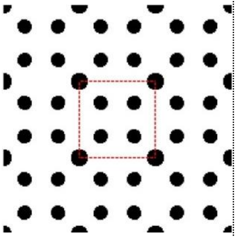	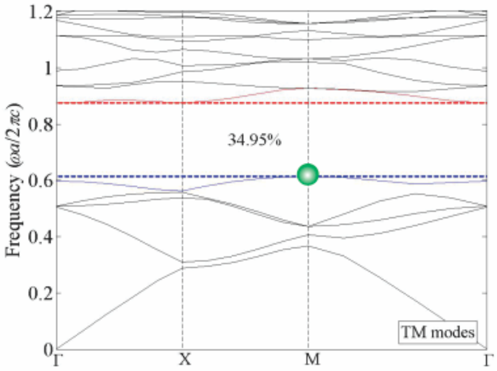
(c)<br>TE-PBG,<br>Band 4 at<br>Γ,<br>Hz field	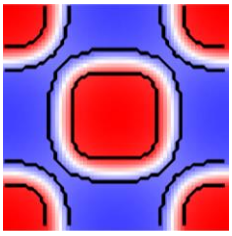	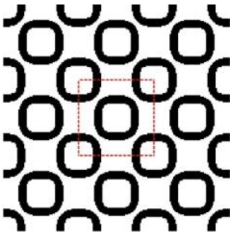	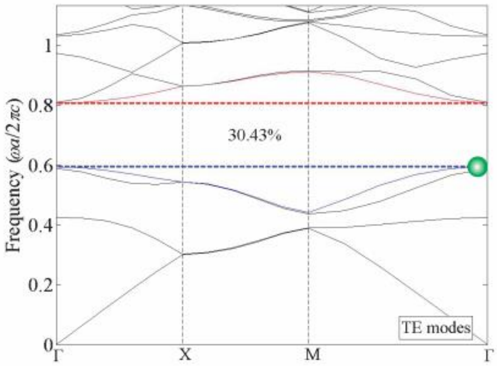
(d)<br>TE-PBG,<br>Band 9 at<br>Γ,<br>Hz field	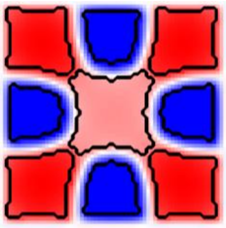	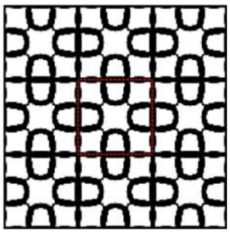	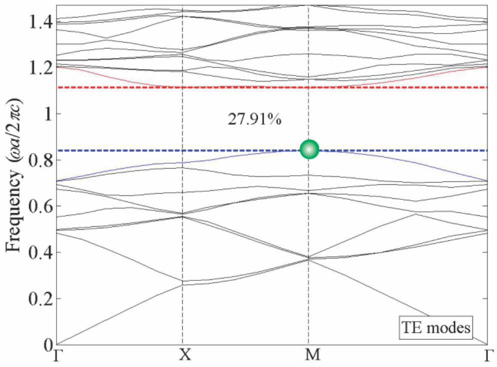

Fig. S1 Bloch modes of high order photonic crystals with ω_{min} at M or Γ. (a)-(b) TM modes with Ez field. (c)-(d) TE modes with Hz field

For the odd number order photonic crystals with a point located at the centre, it is a little ambiguous to count the points because it forms a singularity point. For example TM-PBG above band 9 (**E**z field at Γ is shown in Fig. S2), there are 4 resonators surrounding the centre and all of them have opposite phase with their neighbours, so the field has to be zero at the centre position. In this situation the 'zero centre' is also a point that needs to place a resonator on. This phenomenon results from the rotational symmetry of photonic crystal lattice.

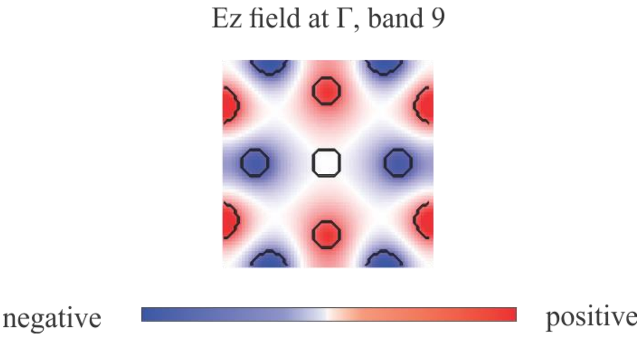

Fig. S2. **E**z field pattern at Γ of TM-PBG N = 9, possessing a singularity point at the centre.

Appendix B: Detailed TM-PBG design

The size of a PBG is influenced by both the spatial distribution and the geometric shape of the points. Former study shows that resonators with a nearly circular shape and reaching a uniform distribution with nearly the same neighbor distance benefit the large TM-PBG (3). Considering the complexity of 2D structures in spatial distributions and geometric shapes, it is impossible to find an analytical solution for the eigenfrequencies in the band diagram and an analytical relationship between the spatial distribution and the geometric shape of the points and the size of the bandgap. However, the effects of the spatial distribution and the shape of the resonators on the gap size can be shown by numerically calculating the gap sizes corresponding to those two factors. Here we use the deviation from the uniform distribution δ and the ellipticity of the ellipse f as variables to quantify their influence on the PBG size. In the followings, we separately analyze the influence of each factor, while maintain the other factor a constant. In this way we are able to clearly show how each factor affects the size of the bandgap.

Take the TM-PBG N=7 as an example. The spatial distribution is quantified by the deviation from the uniform distribution (Equation (3) in the manuscript). For the uniform distribution, δ = 0. The numerical calculation results are shown in the following Fig R2. In this case, the ellipticity of the resonantors remains unchanged. When δ increases, the distribution of the points P_i deviates from the uniform distribution, the gap size decreases significantly because of the weak local confinement of the resonance in each resonator. This diagram shows that the gap size is influenced by the spatial distribution of the points without changing the geometric shape of each resonator.

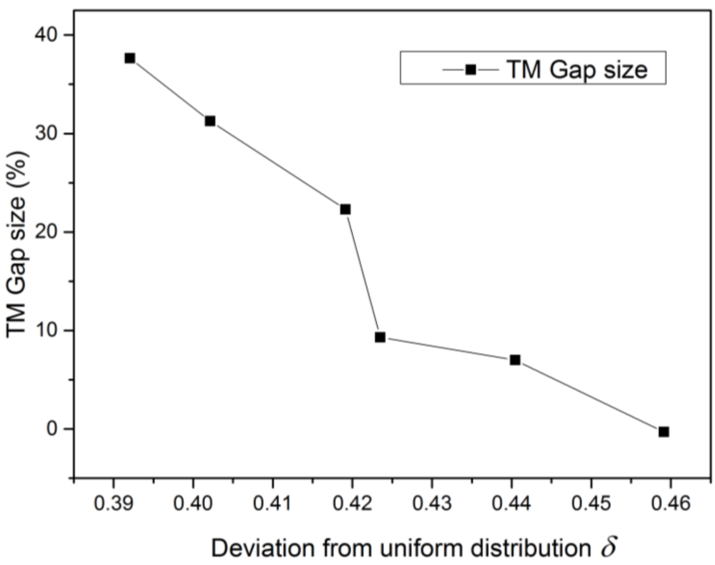

Fig. S3, The relationship between PBG size and the deviation from uniform distribution (TM-PBG, N = 7, ε = 12).

On the other hand, the geometric shape is quantified by the ellipticity of the ellipse $f = B/A$, which is defined as the ratio of two axes of elliptical resonator on the diagonal, as shown in Fig. S5(a). Circular rods with an identical size lead to stronger localized confinement of **E** field thus benefit a large TM-PBG size (3). However, identical circular rods may change the actual lattice constant of the periodic structure and the lattice constant can be further reduced. Therefore it is required to break the transform symmetry within the unit cell without changing the rotational symmetry. In our designs we break the circular symmetry of rods locating on the diagonal lines and adopt elliptical design, so the rotational symmetry remains unchanged. The minor axis of the ellipse $A = r$ in Eq.(4). The major axis of ellipse lies along the diagonal line and the axes relationship is B=1.1A (Fig. S5(a)). The size of the resonator needs to be comparable with wavelength, so A ~ λ. Therefore increasing B at least 0.1A can tell the difference.

The results are shown in Fig. S5(b), in which δ is kept the same. The gap size achieves the maximal at B=1.1A, indicating the shape of the rod is close to a circle. Fig.S5(b) shows that the gap size is influenced by the geometric shape of the resonators without changing the spatial distribution. Similarly, optimal structures supporting large TE-PBGs have connected walls with an identical thickness. The walls are the Voronoi diagram generated by the N points, with the thickness t also determined by Eq. (4).

As a result, we can safely conclude that both the spatial distribution and the geometric shape affect the size of the bandgap. Therefore, those two parameters are carefully considered in our designing process as demonstrated in detail in the manuscript.

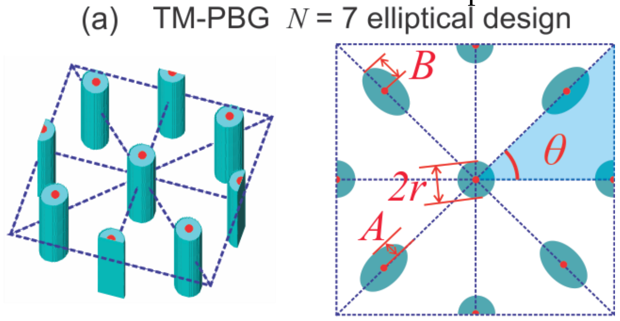

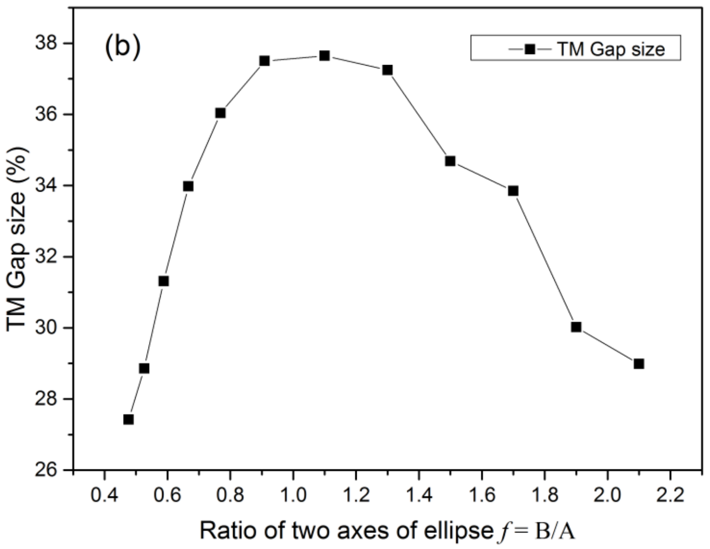

Fig. S4 (a) Ellipse geometry designs on the diagonal lines.(b) The relationship between PBG size and the deviation from circular shape (TM-PBG, N = 7, ε = 12).

Appendix C: Topology of resonator & Global result

In our study, the solid rods/walls without any other topological shape inside mainly support fundamental modes in each resonator, and they form N bands with the lowest energies. At the low frequency bands below the first gap, the periodic structure supports propagating solutions for all frequencies (4). Thus photonic bands below the PBG that will cover the whole frequency range. In this way the generated PBG is the first gap with lowest frequencies.

According to Equation (1), the increase of central frequency ω_0 leads to small PBG size. Resonators with more topological shape (such as holes inside the resonator, Fig.S5 (a)) mainly support high order modes in high frequencies, resulting of PBG with smaller gap size. So the first PBG just above these N bands is the lowest and largest one, which is the global optimal PBG at a given frequency.

Without initial structures as the constraint, the topology optimization possibly generates structures with more features. Fig.S5 shows the results of two different topology optimization processes, both aiming at maximizing the gap between band 3 and band 4. The first one starts with a random structure and the second one starts with initial structure that satisfies our criteria. It is obvious that though other structures can generate a PBG between appointed bands, the PBG is not the first gap thus the size is smaller, which is not the global optimal PBG between given bands.

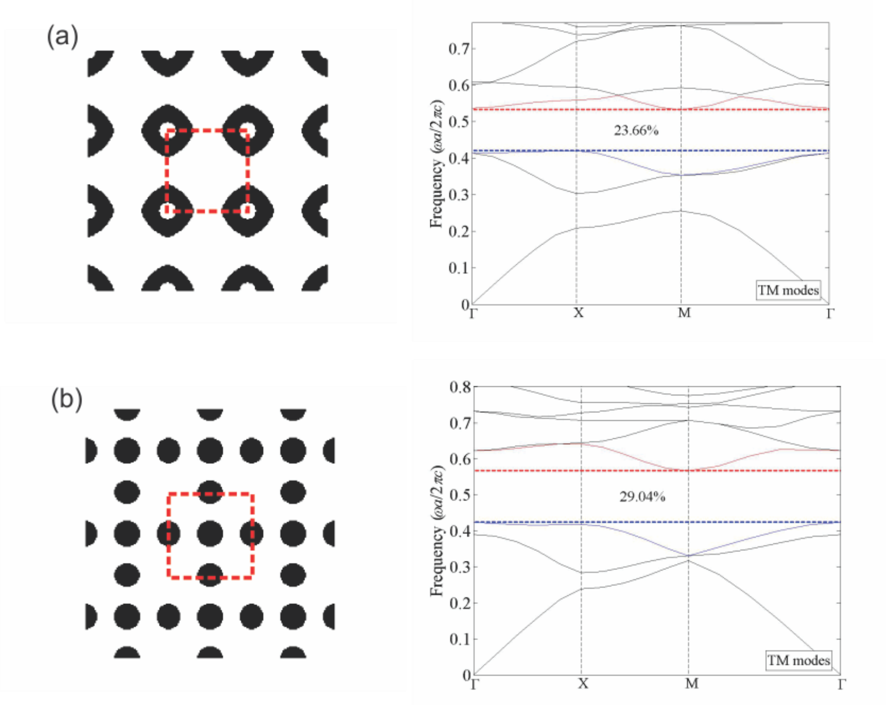

Fig. S5. Topology optimization results. (a) Optimization starts with a random structure. (b) Global optimization results starts with initial structure that satisfies our criteria

Appendix D: The role of topology optimization

Based on Bloch mode analysis, the analytical design shows the topology to achieve global optimal structures. However multiple geometry shapes correspond to one topology. Considering the complexity of resonator geometric shapes, there is no analytical solution for global optimal PBG. Final optimal geometry can be acquired by using topology optimization. Based on the mentioned criteria, we have developed topological optimization method with constrained initial conditions (Fig.S6) (5), aiming at finding the exact topology structure with maximal PBG. The constrained initial structures satisfy our analytical criteria. The process is controlled to change the geometry of each part without changing the topology to achieve global maximum PBG. The small average discrepancy between the designed and the optimized gap sizes (Figs. 3 (a) & (b) in manuscript) indicate our analytical design has achieved nearly optimal results.

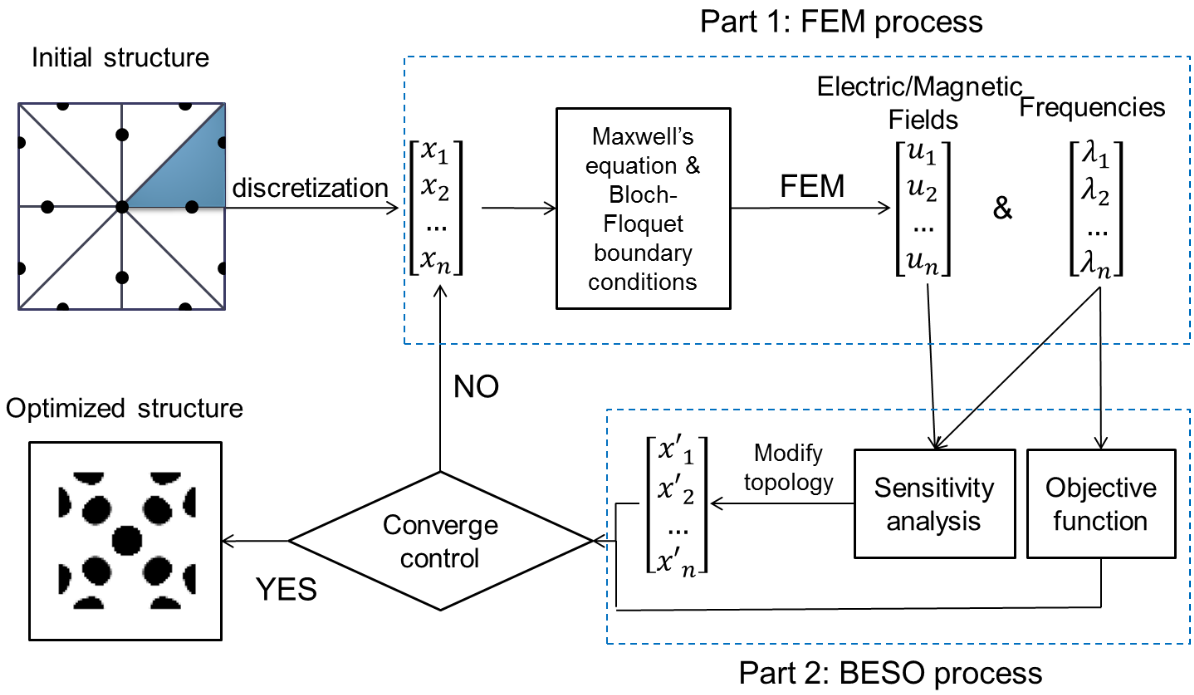

Fig.S6. The process of topology optimization with initial structures

Appendix E: The missing CPBG band order

For a CPBG structure design, if the intersection is formed by four Voronio partitions instead of three (Fig. S7), the **H** field in intersection should be zero since **H** field of TE mode in neighbour partitions has opposite phase. **E** field achieves maximal on the zero-panel of **H** field. Now we assume **E** field achieves positive maximum on the (**+H-H**) panel, and on the (**-H+H**) panel **E** is negative maximum, then the **E** field of intersection also need to be zero at the intersection. That's why it forms a singularity and cannot generate TM mode in this intersection. Therefore the TM-PBG cannot generate at the *appointed order* and the overlap with *appointed* TE-PBG is not available. As a result, some CPBG orders are missing (TE-TM: 1-1, 2-2, 4-4, 8-10 and 9-13).

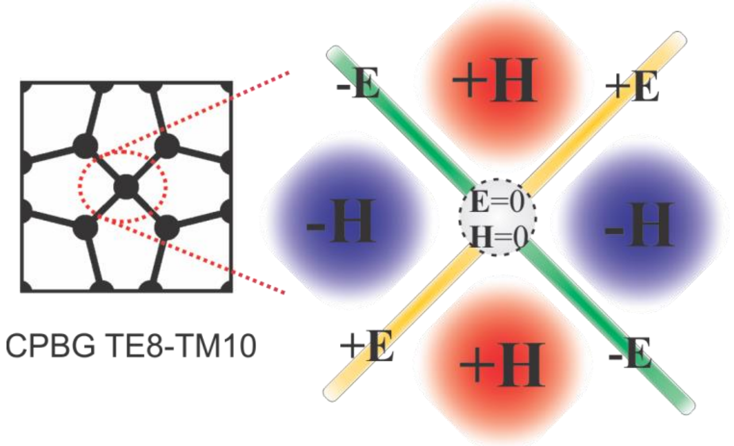

Fig.S7. Schematic of the generation of electromagnetic field singularity.

References

1. Joannopoulos JD, Johnson SG, Winn JN, Meade RD. Photonic crystals: molding the flow of light: Princeton university press; 2011.
2. Baldo M. Introduction to Nanoelectronics. MIT OpenCourseWare, License; 2011.
3. Jia L, Bita I, Thomas EL. Impact of Geometry on the TM Photonic Band Gaps of Photonic Crystals and Quasicrystals. Physical Review Letters. 2011;107(19):193901.
4. Lidorikis E, Sigalas MM, Economou EN, Soukoulis CM. Tight-Binding Parametrization for Photonic Band Gap Materials. Physical Review Letters. 1998;81(7):1405-8.
5. Meng F, Huang X, Jia B. Bi-directional evolutionary optimization for photonic band gap structures. Journal of Computational Physics. 2015;302:393-404.
